# PD165 To What Extent Do Health Technology Assessment Bodies Cross-Reference Each Other In Their Reports?

**DOI:** 10.1017/S026646232400391X

**Published:** 2025-01-07

**Authors:** Peter Wagner, Paula Szawara, Sattwik Kumar Panda, Vinay M Kanthi

## Abstract

**Introduction:**

Due to different timing of drug launches across countries, published health technology assessment (HTA) findings from one country may impact HTA outcomes in other countries. The aim of our work was to identify the most influential HTA bodies by analyzing to what extent HTA bodies cross-reference each other in their HTA reports.

**Methods:**

We analyzed the HTA reports on single drug assessments (SDA) published by 46 HTA bodies from 28 countries (and cross-country collaborations) with decision dates between January 2011 and November 2023. We searched the identified HTA reports by using natural language processing and a predefined set of keywords to identify whether, and to what extent, HTA bodies reference each other in their HTA reports. Additionally, we assessed if there is a trend over time in the cross-referencing, and whether any clusters could be identified.

**Results:**

Based on the analysis of 24,793 SDAs, the National Institute for Health and Care Excellence (NICE) was referenced the most (in 4,198 HTA reports across 39 HTA bodies), followed by the Canadian Agency for Drugs and Technologies in Health (in 2,034 reports across 35 HTA bodies), and the Scottish Medicines Consortium (SMC) (in 1,960 reports across 31 HTA bodies). The HTA bodies that most often referenced other HTAs were the Agency for Health Technology Assessment and Tariff System, the Haute Autorité de santé, and NICE. Seven HTA bodies were not referenced in any HTA report, while four did not reference any other HTA body.

**Conclusions:**

Our research shows that most of the analyzed HTA agencies not only referenced other HTA bodies in their HTA reports but were also referenced by other HTA bodies. The most often referenced HTA agencies were mostly from English-speaking countries, were well recognized, and had well defined methodologies.

